# Supplementary material for: Symptomatic malaria enhances protection from reinfection with homologous Plasmodium falciparum parasites
Source: PLoS Pathog. 2023 Jun 12;19(6):e1011442. doi: 10.1371/journal.ppat.1011442 (PMC10289385; doi:10.1371/journal.ppat.1011442)
Supplement: S1 Text — Table A. Comparison of index infection episodes included and excluded from survival analyses. Table B. Cox mixed effect model estimates for reinfection with parasites bearing homologous epitope types after symptomatic and asymptomatic exposure, without and with adjustment for MOI. Fig A. Comparative diversity of nucleotide haplotypes and epitope types. Fig B. Comparison of epitope type frequencies in index infections with time to reinfection less than or greater than 60 days. Fig C. Risk of reinfection with P. falciparum parasites bearing homologous epitopes. Fig D. Time to reinfection with random epitope type stratified by symptomaticity. Fig E. Analyses of parasites defined by CSP DV10. Fig F. Analyses of parasites defined by combined CSP-Th2R/Th3R epitopes. (DOCX) [file ppat.1011442.s001.docx]

**Symptomatic malaria enhances protection from reinfection with homologous *Plasmodium falciparum* parasites**

***Supplemental Information***

**Supplemental Results**

**Analysis of CSP DV10 positions**

When classified by CSP-DV10 epitope type, we observed 897 type infection episodes.The probability of homologous CSP-DV10 epitope type reinfection within 1 year was 0.71 (95% CI 0.67 – 0.75), similar to reinfection with a random epitope type (log-rank median chisq 0.661, p = 0.416) (**Fig E panel A in S1 Text**). In randomized datasets, there was no difference in the risk of homologous reinfection between symptomatic and asymptomatic infections (**Fig E panel B in S1 Text**; log-rank test median chisq 0.425, p = 0.514). There was not a statistically significant decrease in the hazard of reinfection with parasites bearing homologous CSP-DV10 epitope types following symptomatic compared to asymptomatic index episodes; however, the estimated adjusted hazard ratio (aHR 0.75, 95% CI 0.53 – 1.05, p = 0.098) followed similar trends to the CSP-Th2R, CSP-Th3R, and AMA-1 c1L epitopes (**Fig E panel C in S1 Text)**.

**Ensemble analysis of CSP Th2R and Th3R positions**

We re-classified parasites on the basis of the 4 most informative amino acid positions in each of the CSP-Th2R and the -Th3R epitopes (**Fig 3)** yielding 84 unique CSP-Th2R/Th3R types. As with prior classifications using only either epitope, the MOI expressed by these epitopes was highly correlated with the *pfcsp* nucleotide sequence (**Fig F panel A in S1 Text**). The probability of homologous CSP Th2R/Th3R epitope type reinfection within 1 year was 0.41 (95% CI 0.38 – 0.44), which was similar to reinfection with a heterologous parasite in randomized datasets (log-rank median chisq 1.53, p=0.217) (**Fig F panel D in S1 Text**), in which there was no difference in the hazard of homologous reinfection between symptomatic and asymptomatic infections (median aHR 1.02; 95% CI 0.79 – 1.32) (**Fig F panel C in S1 Text**). However, similar to the associations observed for parasites defined on either CSP Th receptor epitope, compared to asymptomatic index infections, the hazard of reinfection with parasites bearing a homologous CSP Th2R/Th3R epitope type following a symptomatic infection was decreased by 44% (aHR 0.56; 95% CI 0.40 – 0.80; p=0.002) (**Fig F panel B in S1 Text**).

**Table A**. **Comparison of index infection episodes included and excluded from survival analyses**

|  | **Total** | **Included** | **Excluded** | **p-value*** |
| --- | --- | --- | --- | --- |
|  |  |  |  |  |
| Time to censoring, mean, days | 99.94 | 137.56 | 37.43 | 2.2e-16 |
| **Type, no. (%)** |  |  |  |  |
| Asymptomatic | 415 (75.3) | 275 (79.9) | 140 (67.6) | 0.0012 |
| Symptomatic | 136 (24.7) | 69 (20.1) | 67 (32.4) |  |
| **Age, no. (%)** |  |  |  |  |
| < 5y | 84 (15.3) | 52 (15.2) | 32 (15.6) |  |
| 5-15 | 228 (41.6) | 129 (37.6) | 99 (48.3) | 0.0270 |
| >15y | 236 (43.1) | 162 (47.2) | 74 (36.1) |  |
| **Sex, no. (%)** |  |  |  |  |
| Female | 315 (57.2) | 194 (56.4) | 121 (58.5) | 0.636 |
| Male | 236 (42.8) | 150 (43.6) | 86 (41.5) |  |
| **Transmission season, no. (%)**** |  |  |  |  |
| High | 268 (48.6) | 148 (43.0) | 120 (58.0) | 0.001 |
| Low | 283 (51.4) | 196 (57.0) | 87 (42.0) |  |

Infections were excluded from survival analyses if reinfection occurred ≤ 60d following the end of the index infection.

* Computed by either the chi-square test or t-test.

** Classified on the basis of the abundance of mosquitos collected in the prior two weeks into high (> 50) or low (≤ 50).

**Table B. Cox mixed effect model estimates for reinfection with parasites bearing homologous epitope types after symptomatic and asymptomatic exposure, without and with adjustment for MOI.**

|  | Main model (no MOI)  Adj. HR (95% CI) | Model with continuous MOI  Adj. HR (95% CI) | Model with categorical MOI  Adj. HR (95% CI) |
| --- | --- | --- | --- |
| **Epitope** | | | |
| **CSP Th2R** | | | |
| **Symptomatic infection** | **0.63 (0.45 – 0.89)** | **0.60 (0.45 – 0.84)** | **0.61 (0.43 – 0.85)** |
| Mosquitoes > 50 | 1.62 (1.33 – 1.98) | 1.61 (1.31 – 1.96) | 1.62 (1.33 – 1.98) |
| Type prevalence | 1.22 (1.19 – 1.25) | 1.22 (1.19 – 1.25) | 1.22 (1.19 – 1.25) |
| Male | 1.05 (0.71 – 1.55) | 1.05 (0.71 – 1.55) | 1.04 (0.70 – 1.53) |
| Age 5-15 | 1.48 (0.80 – 2.76) | 1.45 (0.78 – 2.69) | 1.46 (0.79 – 2.69) |
| Age > 15 | 0.99 (0.53 – 1.82) | 0.97 (0.52 – 1.79) | 0.98 (0.53 – 1.80) |
| MOI (continuous) | --- | 0.97 (0.95 – 1.00) | --- |
| MOI 2-5 | --- | --- | 1.20 (0.86 – 1.67) |
| MOI > 5 | --- | --- | 0.91 (0.64 – 1.28) |
| AIC | 611.00 | 612.15 | 611.76 |
| LR test, p-value* |  | 0.1263 | 0.0676 |
| **CSP Th3R** | | | |
| **Symptomatic infection** | **0.71 (0.52 – 0.97)** | **0.68 (0.49 – 0.93)** | **0.71 (0.52 – 0.97)** |
| Mosquitoes > 50 | 1.36 (1.10 – 1.69) | 1.36 (1.10 – 1.68) | 1.36 (1.10 – 1.69) |
| Type prevalence | 1.13 (1.11 – 1.14) | 1.12 (1.11 – 1.14) | 1.13 (1.11 – 1.14) |
| Male | 1.13 (0.80 – 1.60) | 1.14 (0.81 – 1.61) | 1.13 (0.81 – 1.60) |
| Age 5-15 | 1.42 (0.83 – 2.42) | 1.39 (0.81 – 2.38) | 1.40 (0.82 – 2.40) |
| Age > 15 | 0.91 (0.54 – 1.56) | 0.90 (0.52 – 1.53) | 0.91 (0.54 – 1.55) |
| MOI (continuous) | --- | 0.96 (0.90 – 1.01) | --- |
| MOI 2-5 | --- | --- | 1.01 (0.76 – 1.35) |
| MOI > 5 | --- | --- | 0.93 (0.65 – 1.33) |
| AIC | 411.60 | 412.99 | 407.98 |
| LR test, p-value* |  | 0.1934 | 0.8630 |
| **AMA-1 c1L** | | | |
| **Symptomatic infection** | **0.63 (0.43 – 0.94)** | **0.54 (0.37 – 0.81)** | **0.56 (0.37 – 0.84)** |
| Mosquitoes > 50 | 1.35 (1.04 – 1.75) | 1.36 (1.06 – 1.76) | 1.40 (1.08 – 1.81) |
| Type prevalence | 1.16 (1.13 – 1.18) | 1.15 (1.13 – 1.18) | 1.16 (1.13 – 1.18) |
| Male | 1.10 (0.72 – 1.69) | 1.06 (0.69 – 1.64) | 1.01 (0.65 – 1.59) |
| Age 5-15 | 1.86 (0.96 – 3.56) | 1.77 (0.91 – 3.44) | 1.78 (0.90 – 3.53) |
| Age > 15 | 1.16 (0.60 – 2.23) | 1.14 (0.58 – 2.22) | 1.15 (0.58 – 2.30) |
| MOI (continuous) | --- | 0.92 (0.87 – 0.97) | --- |
| MOI 2-5 | --- | --- | 1.40 (0.97 – 1.97) |
| MOI > 5 | --- | --- | 0.74 (0.50 – 1.10) |
| AIC | 355.66 | 368.29 | 377.64 |
| LR test, p-value* |  | 0.0028 | 0.0003 |

* Relative to “Main model (no MOI)”

AIC: Akaike information criterion; LR: likelihood ratio

**Supplemental Figures**


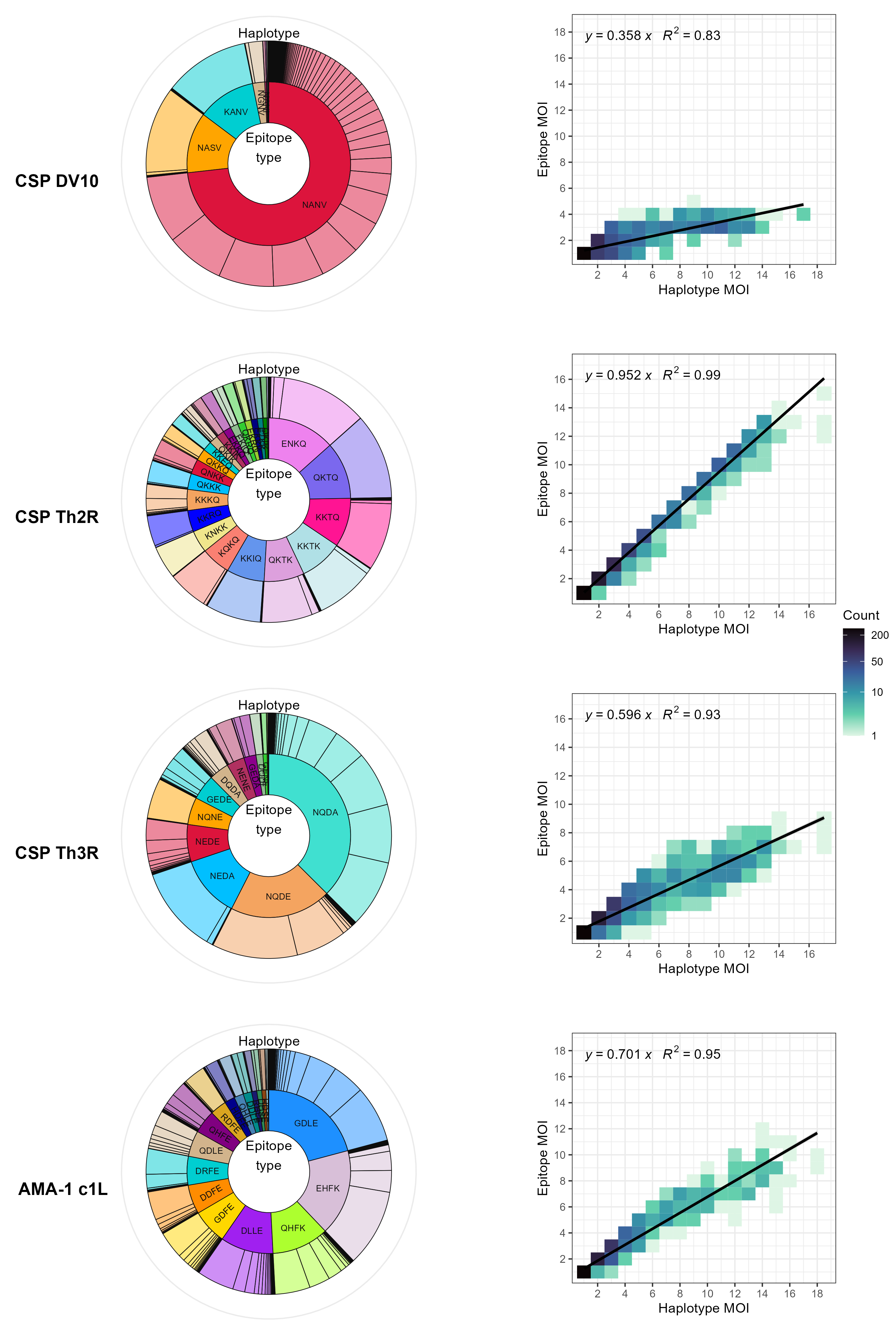


**Fig A. Comparative diversity of nucleotide haplotypes and epitope types.** Left: Sunburst plots displaying haplotypes (outer circle) defined on unique nucleotide sequences of each fragment and corresponding epitope types (inner circle) for each protein fragment defined by the four most important amino acid positions observed in the CSP DV10 epitope (amino acid positions 298, 299, 301, 303, n = 861 samples), CSP-Th2R epitope (amino acid positions 318, 321, 322, 324, n = 861 samples), CSP-Th3R epitope (amino acid positions 352, 357, 359, 361, n = 861 samples), and AMA-1 c1L (amino acid positions 197, 200, 201, 206, n = 724 samples). Right: Plots of the correlation between multiplicity of infection (MOI) defined by nucleotide haplotype and by epitope type defined by the four most important amino acid positions observed in the CSP DV10 epitope, CSP Th2R epitope, CSP Th3R epitope, and AMA-1 c1L domain. Color indicates the number of samples within each bin, and lines are regression output.


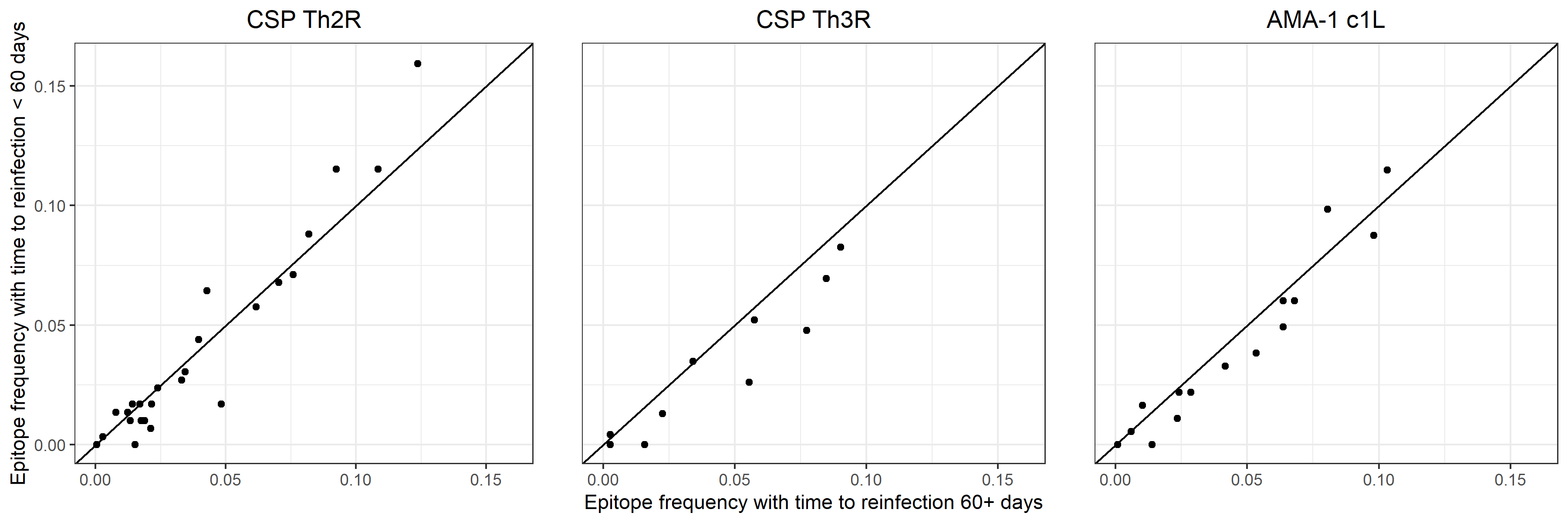


**Fig B. Comparison of epitope type frequencies in index infections with time to reinfection less than or greater than 60 days.** Each dot indicates the prevalence of a unique CSP-Th2R (left), CSP-Th3R (middle), or AMA-1 c1L (right) type in index infections with (y-axis) or without (x-axis) reinfection within the first 60 days following clearance. Black line indicates X=Y.

**Fig C. Risk of reinfection with *P. falciparum* parasites bearing homologous epitopes**. Kaplan-Meier survival curves of time to homologous type reinfection defined by the CSP-Th2R epitope (top), CSP-Th3R epitope (middle), or the AMA-1 c1L epitope type (bottom) stratified on the observed dataset (grey) or randomized datasets (black) after all index episodes (left), asymptomatic index episodes (middle) and symptomatic index episodes (right). Each unique type present in a sample was considered an index case when the type was absent in the subsequent sample. Reinfection was defined as first sample harboring the same specific type. Time to reinfection with a random type was determined by permuting epitope types at the sample level and then treated as the time to homologous reinfection. Survival curves for reinfection with random epitopes represent the median survival probabilities and confidence intervals of 1000 permuted datasets. Infections that were not followed by a reinfection were censored at the end of the study period and are denoted by crosses. Vertical dotted line indicates the start of the defined time at risk of re-infection, and shaded area shows the 95% CIs.


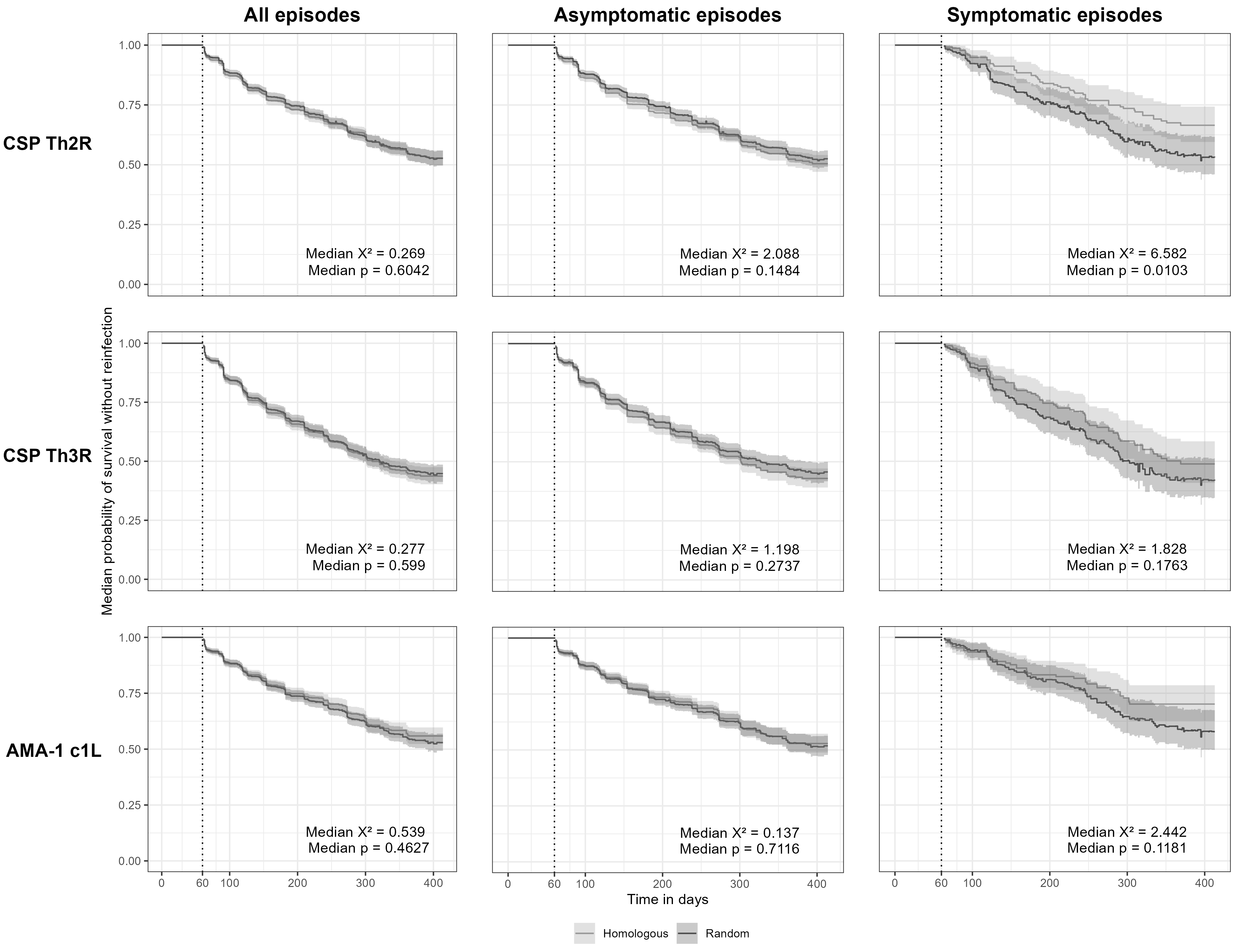

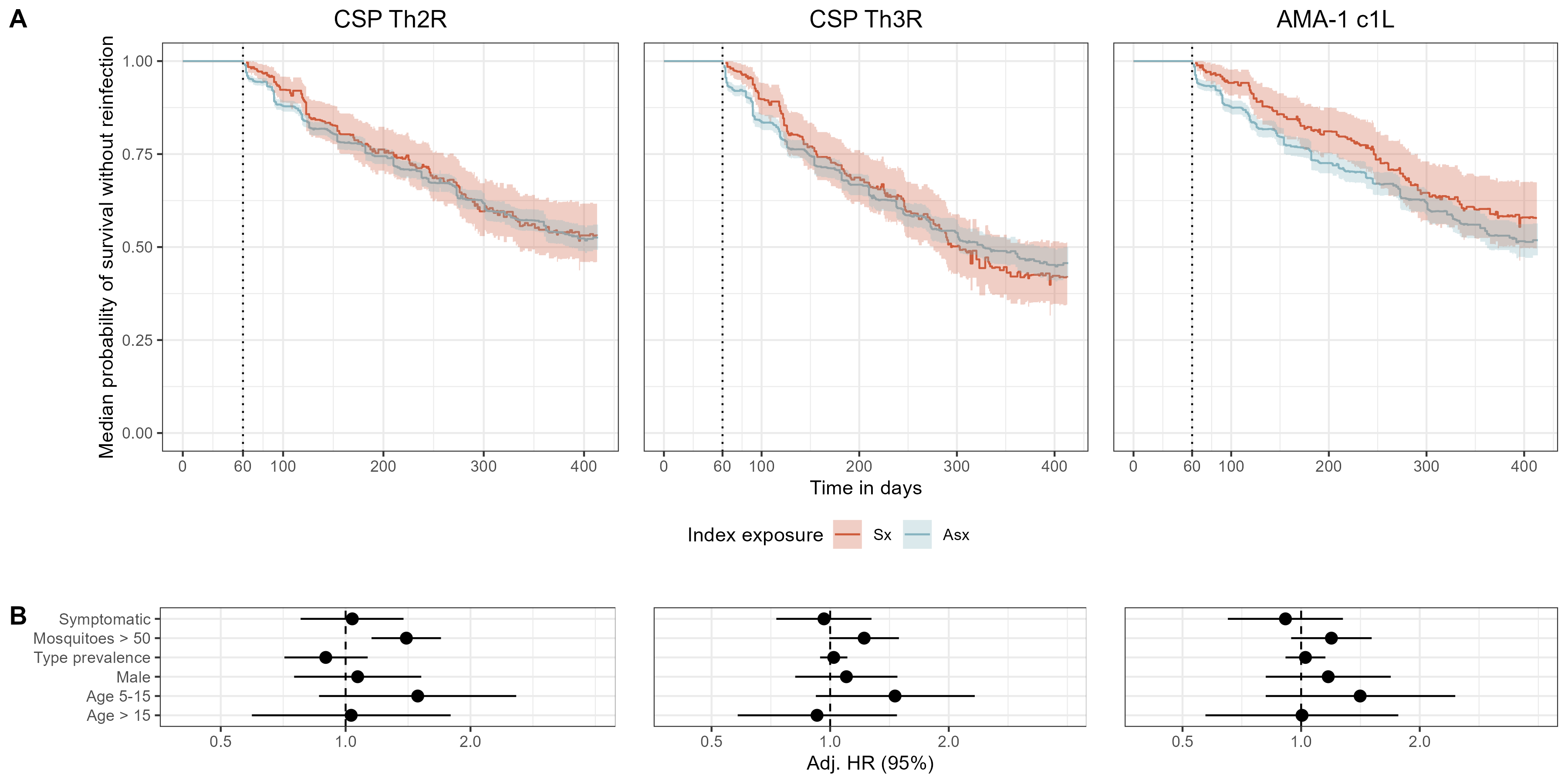


**Fig D. Time to reinfection with random epitope type stratified by symptomaticity. A)** Kaplan-Meier survival curves of time to reinfection by parasites with random CSP-Th2R type, CSP-Th3R type, or AMA-1 c1L types. Randomized datasets (1000) were generated by permuting epitope types at the sample level, and median survival probabilities are plotted. For each infection and each antigen, each unique type is treated as a separate unique infection. Curves are stratified by index case being either asymptomatic (Asx) or symptomatic (Sx). Infections that were not followed by a reinfection were censored at the end of the study period. Shaded areas indicate the median 95% confidence interval (CI). **B)** Median mixed effects Cox proportional hazards model results for reinfection with homologous CSP Th2R epitope types, homologous CSP Th3R epitope types, and homologous AMA-1 c1L types. Circles indicate the median adjusted Hazard Ratio (HR); lines show the median 95% confidence interval. Adj. HR, adjusted hazard ratio.


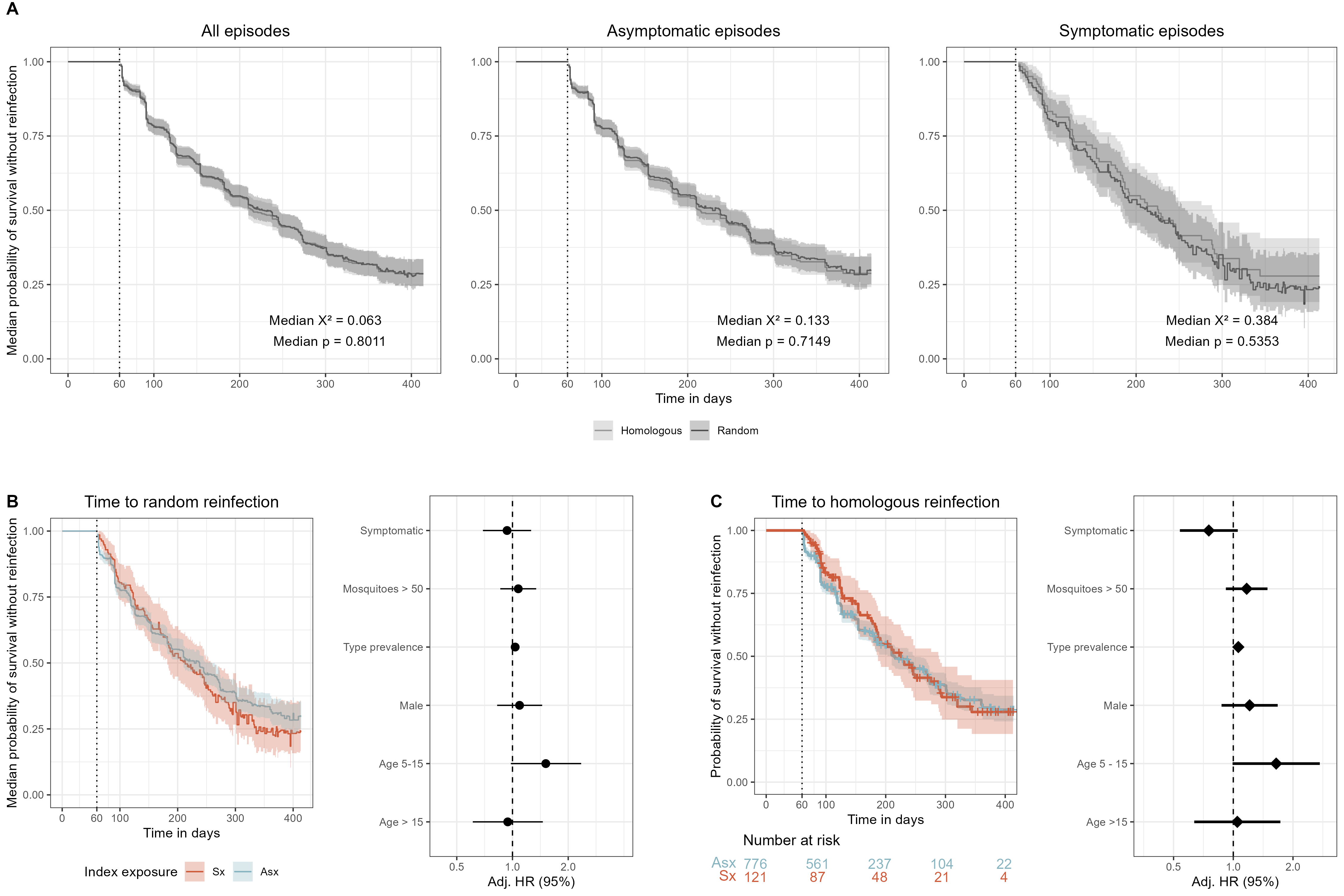


**Fig E. Analyses of parasites defined by CSP DV10. A)** Kaplan-Meier survival curves of time to homologous type reinfection defined by the CSP-DV10 epitope stratified on the observed dataset (grey) or randomized datasets (black) after all index episodes (left), asymptomatic index episodes (middle) and symptomatic index episodes (right). **B)** Kaplan-Meier survival curves of time to reinfection by parasites with random CSP-DV10 types after asymptomatic index episodes (blue) and symptomatic epidodes (red) and median mixed effects Cox proportional hazards model results for reinfection with random CSP-DV10 epitope types. Circles indicate the median adjusted Hazard Ratio (HR). **C)** Kaplan-Meier survival curves of time to reinfection by parasites with homologous CSP-DV10 types after asymptomatic index episodes (blue) and symptomatic epidodes (red) and mixed effects Cox proportional hazards model results for reinfection with homologous CSP-DV10 epitope types. Diamonds indiciate adjusted Hazard Ratio (HR); lines show the median 95% confidence interval. Adj. HR, adjusted hazard ratio.


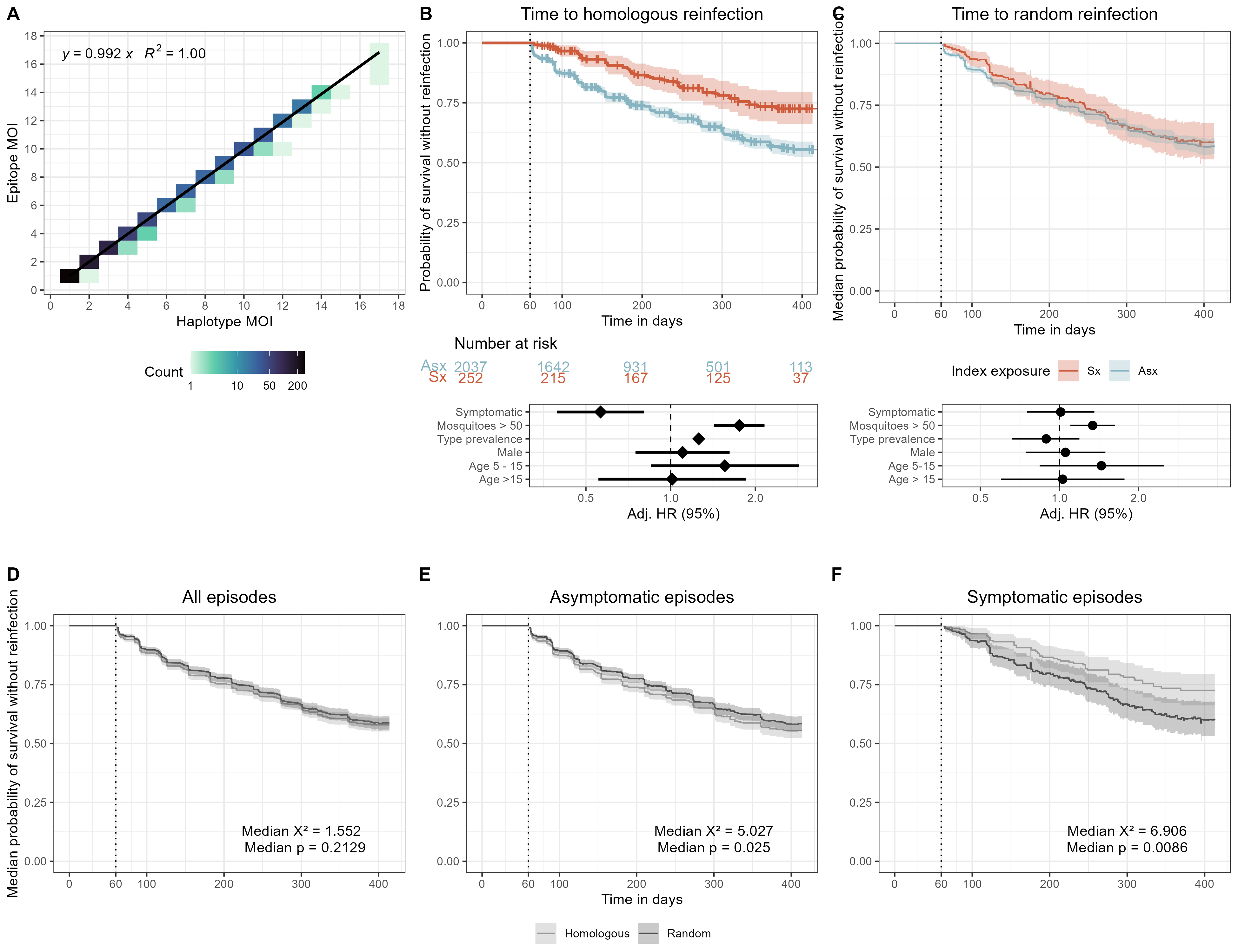


**Fig F. Analyses of parasites defined by combined CSP-Th2R/Th3R epitopes. A)** Plot of the correlation between multiplicity of infection (MOI) defined by nucleotide haplotype and by epitope type defined by the 8 most important amino acid positions observed in the combined CSP-Th2R/Th3R epitope. Color indicates the number of samples within each bin, and lines are regression output. **B) and C)** Kaplan-Meier survival curves of time to reinfection by parasites with homologous combined CSP-Th2R/Th3R type **(B)** or random combined CSP-Th2R/Th3R type **(C).** Curves are probability stratified by index case as either asymptomatic (Asx, blue) or symptomatic (Sx, red). Table shows the number of participants at risk stratified by symptomaticity of index infection at day 0, 100, 200, 300, and 400. For **(C)**, Randomized datasets (1000) were generated by permuting epitope types at the sample level, and median survival probabilities are plotted. Adjusted Hazard Ratios estimated by multivariate mixed effects Cox proportional hazards models for reinfection by parasites bearing homologous CSP-Th2R/Th3R epitopes **(B)** or random CSP-Th2R/Th3R epitopes **(C).** For survival curves, crosses indicate infections that were not followed by a reinfection with a homologous parasite type and therefore censored at the end of the study period, vertical dotted line indicates the start of the defined time at risk of re-infection, and shaded areas indicate the 95% confidence interval. Diamonds indicate adjusted HRs, and bars show the 95% CIs. Adj. HR, adjusted hazard ratio. **D), E), F)** Kaplan-Meier survival curve of time to homologous type reinfection defined by the CSP-Th2R/Th3R epitope stratified on the observed dataset (grey) or randomized datasets (black) after all index episodes **(D)**, asymptomatic index episodes **(E)**, and symptomatic episodes **(F)**. Time to reinfection with a random type was determined by permuting epitope types at the sample level and then treated as the time to homologous reinfection. Survival curves for reinfection with random epitopes represent the median survival probabilities and confidence intervals of 1000 permuted datasets. Crosses indicate infections that were not followed by a reinfection with a homologous parasite type and therefore censored at the end of the study period. Vertical dotted line indicates the start of the defined time at risk of re-infection, shaded areas indicate the 95% confidence interval.
