# Supplementary material for: Symptomatic malaria enhances protection from reinfection with homologous Plasmodium falciparum parasites
Source: PLoS Pathog. 2023 Jun 12;19(6):e1011442. doi: 10.1371/journal.ppat.1011442 (PMC10289385; doi:10.1371/journal.ppat.1011442)
Supplement: Attachment — Submitted filename: Mozzietopes PlosPathogens Rev1Responses v1.docx [file ppat.1011442.s002.docx]

**Department of Medicine**

**Division of Infectious Diseases**

2 May 2023

Kasturi Haldar PhD

Michael Malim PhD

Editors-in-Chief, *Plos Pathogens*

Drs Haldar & Malim:

This letter accompanies the re-submission of our manuscript entitled “Symptomatic malaria enhances protection from reinfection with homologous *Plasmodium falciparum* parasites.” We appreciate the chance to respond to the thoughtful comments of your reviewers.

My co-authors Christine Markwalter, Jens Petersen, Erica Zeno, Kelsey Sumner, Elizabeth Freedman, Judith Mangeni, Lucy Abel, Andrew Obala, and Wendy Prudhomme-O’Meara approved submission of this version of the manuscript.

Below we have enumerated our point-by-point responses to the reviewer comments. For each comment, the reviewer comments are italicized, and our responses are indented and in normal text, with specific text changes underlined.

We’ve included tracked and clean versions of the revised text. Because we have not made changes to the Supplement, there is no tracked version of that document.

**Reviewer #1**

1. *Authors in the introduction frame the data by suggesting that symptomatic malaria infection is associated with induction of more robust immunity compared to asymptomatic infections. However, I believe that causality has been miss-interpreted in this framing. While the literature does show that symptomatic infection is associated with high levels of inflammatory responses, this should not be interpreted as asymptomatic infections being ‘low immune induces’. Indeed, the presences of an asymptomatic infection is likely due to the already existing, prior establishment of protective immune responses. This prior immunity is then able to control parasite density and/or limit immune pathology. Consistent with protective prior immunity in asymptomatic cases in the authors data set is the significantly lower parasite burden in asymptomatic infections.*

RESPONSE: As the reviewer points out, the features of immune responses to malaria parasites are different between symptomatic and asymptomatic infections. We have in general softened our language in the abstract and introduction to reflect this:

Line 31: “Symptomatic malaria ~~more effectively promotes functional immune responses~~ provides more durable protection against reinfection with parasites bearing homologous epitope types.”

Line 45: “The association of symptomatic malaria with ~~enhanced protective immunity~~ delayed homologous reinfection offers a signature of ~~functional immunity~~ protection by which to identify novel targets of antiparasite immunity.”

Line 75: Because features of immune responses to *P. falciparum* differ between symptomatic malaria, which is associated with overexpression of pro-inflammatory cytokines IFN-γ, TNF, and IL-1β,^14^ and asymptomatic infections, we explored if symptomaticity influenced the risk of reinfection with parasites bearing homologous CSP and AMA-1 epitope types. Given that symptomatic infection is associated with the subsequent development of more robust functional immune response for other pathogens such as SARS-CoV-2,^16^ we hypothesized that, compared to asymptomatic infections, symptomatic malaria would prolong the time to reinfection with parasites bearing homologous CSP and AMA-1 types.

We have additionally added the following text to the discussion:

Line 343: “Finally, an important limitation of this study is that it does not explore the protection conferred by asymptomatic infection relative to the absence of an index infection. Asymptomatic infections likely result from prior establishment of protective immune responses that control parasite density and/or disease (Struik and Riley Immunological Reviews 2004, Rodriguez-Barraquer et al. eLife 2018, Stanisic et al Infection and Immunity 2009), and they also can lead to humoral immune responses and protection (Ofori et al Infection and Immunity 2002, Wu et al BMC Medicine 2020, Markwalter et al Malaria Journal 2021). Our observations are not incompatible with existing insights into the interplay between asymptomatic infection and immunity, but rather provide evidence that symptomatic episodes lead to more durable protection against homologous reinfection than do asymptomatic infections.”

2. *While there is some literature in other infections and vaccinations that symptoms can be associated with induction of protective immunity to the best of my knowledge evidence for this in malaria is lacking. At the very least, the authors do not provide sufficient citation of prior literature that would support this hypothesis in malaria in the introduction.

   Introduction line 73-75 – these statements sound like the authors are proposing that symptomatic malaria results in better immunity induction compared to asymptomatic infection. However Ref 15 Portugal et al, doenst support this concept, and only looks at symptomatic malaria, not compared to asymptomatic malaria in comparison. This citation is also used to say that febrile malaria resulted in upregulation of multiple markers of adaptive immunity – which is true, but that doesn’t mean that asymptotic infection doesn’t result in increased immune development.

   Statement line 174: Presence of symptoms during an index infection reflects of immune response to the parasite, we next…*

RESPONSE: We edited lines 73-75 (see #1 above) to remove the implication that symptomatic infections lead to more robust immune responses than asymptomatic infections.

Line 174: “Given the rationale that symptomatic malaria and asymptomatic infections lead to distinct immune responses to the parasite, we next…”

3. *A symptomatic infection, with high parasite infection is indicative of uncontrolled parasite growth, and while inflammation is indicative of an active immune response, that is not directly linked to the development of a protective adaptive response – aka inflammation doesn’t necessarily mean robust development of protection. If the authors know of data that does support this hypothesis, these papers need to be specifically cited. I interpret the existing data in the opposite direction, an asymptotic infection is low density, indicating a adaptive response that exists already and can control the parasite. Authors need to consider whether there is any existing data from the malaria immunity space that supports their framing and hypothesis sufficiently. For example, there is data on the other side – that parasite density doesn’t associated with antibody development (see Chan et al, CRM, 2020), and that antibodies are induced in asymptomatic infection (for example in Pv, but similar concept Longley, Malaria Journal, 2017).*

RESPONSE: We agree that an asymptomatic infection likely indicates pre-existing immunity that controls parasite density. We also agree that low-density and asymptomatic episodes can induce antibody responses. We also believe that neither of these observations is incompatible with our rationale/hypothesis that the immune response after a symptomatic episode is different from that generated by an asymptomatic episode. As detailed in #1 and #4 below, we have added text to the Introduction and Discussion to address these specific points.

4. *Along with the framing/hypothesis underpinning the paper, the discussion and interpretation of the results needs to be carefully re-considered. Specifically, the authors do not discuss evidence that symptomatic blood stage infection disrupts immunity to pre-erythrocytic stages. See lines 244 and section 289-302. Authors need to discuss the data of doi: 10.1016/j.celrep.2016.11.060 DOI: 10.1371/journal.ppat.1009594. Both of these studies clearly show that blood stage infection (where symptoms occur) disrupt immunity to the sporozoite stage (CSP). As such, the hypothesis/rational for the symptomatic impact of improving immunity is not at all supported by these studies.*

RESPONSE: We have added the text below to the Discussion describing the interplay between blood and liver stage infections, immunity, and symptomaticity:

LINE 304: “However, there is clear interplay between blood- and liver-stage infection and immunity, as existing blood stage infection disrupts the immune response to concurrent pre-erythrocytic infection or vaccination with attenuated sporozoites (Keitany et al 2016 Cell Reports; Murphy et al 2021 Plos Pathogens). Relevant to our findings is the evidence that exposure to pre-eryothrocitic parasites modulates the response and severity of blood stage infections. For example, liver stage activation of distinct γδ T cell subsets protects against severe disease during the blood stage in mouse models (Chora et al. 2023 Immunity). Additionally, given their critical role in pre-erythrocytic immunity,^26,27^ effector CD8+ T-cells primed by CSP exposure may be converted more efficiently…”

5. *Line 262 – the greatest protection following symptomatic infection among the rarest types than the most common types. This suggests that the rarity of these epitope types may be a consequence of the protection conferred by symptomatic infections harbouring them, and highlights a mechanisms by which anti-parasite immunity shapes parasite diversity. Again, authors need to carefully re-consider causality in their framing and interpretation of the data. Asymptomatic infection may here be due to prior exposure and pre-existing immunity to existing variants.*

RESPONSE: We agree that asymptomatic infection may be the result of prior exposure and preexisting immunity to existing variants. We have added a sentence reflecting that this may be the underlying reason for the observed effect modification:

Line 269: “This suggests that the rarity of these epitope types may be a consequence of the protection conferred by symptomatic infections harboring them, and we hypothesize that anti-parasite immunity may shape parasite diversity. Alternatively, compared to common epitopes, these rare epitopes may be more likely to cause symptomatic episodes owing to a lower background of exposure across the population.”

**Reviewer #2**

6. *I have one major suggestion with the analysis. 1) Asymptomatic infections are more likely to be persistent, and a higher detection of homologous reinfections following asymptomatic infections could represent misclassification of persistent infections that transiently escape the limit of detection as new infections. Although the authors do note this in the limitations section of the discussion, could the authors perform an additional sensitivity analysis to define the end of infection as the absence of the parasite or type in the subsequent 2 visits? This would help with interpretation of the results, in my opinion.*

RESPONSE: We performed a sensitivity analysis requiring two parasite-negative samples to define the end of an episode, to mitigate the risk that an observed episode and subsequent reinfection was really a persistent infection with parasite densities that transiently dipped below detectable levels. As expected, applying this stringent definition reduced the numbers of index infections and of reinfections. Additionally, the monthly active sampling scheme results in an increase in the effective floor from 60 to 90 days for reinfection after an asymptomatic episode. Because this floor is not present for passively detected symptomatic infections, we then excluded index episodes for which time to reinfection was less than or equal to 90 days from the sensitivity analysis.

The results are below in **Table R1**. As expected, the decreased number of index and reinfections led to wider confidence intervals; however, the direction of the adjusted hazard ratios remained consistent between the main and sensitivity analysis, providing reassurance that our findings were likely not biased by the episode definition. We have not added this sensitivity analysis to the manuscript due to the large set of existing analyses in both the text and the supplement. But, we’d be happy to shoehorn it in somehow, probably also in the supplement, if yall feel it would add additional value for a reader.

**Table R1: Comparison of mixed effect models with the original classification**

|  | Main analysis | Sensitivity analysis |
| --- | --- | --- |
| Index events (N) | | |
| Overall | 344 | 239 |
| Th2R | 2174 | 1816 |
| Th3R | 1462 | 1196 |
| c1L | 1194 | 1123 |
| Reinfections observed (N) | | |
| Overall | 241 | 159 |
| Homologous Th2R | 687 | 492 |
| Homologous Th3R | 565 | 403 |
| Homologous c1L | 400 | 274 |
| Adjusted Hazard Ratio of reinfection [Sx vs Asx] aHR (95% CI) | | |
| Overall | 1.04 (0.74 – 1.46) | 1.30 (0.84 – 2.02) |
| Homologous Th2R | 0.63 (0.45 – 0.89) | 0.81 (0.55 – 1.19) |
| Homologous Th3R | 0.71 (0.52 – 0.97) | 0.92 (0.64 – 1.32) |
| Homologous c1L | 0.63 (0.43 – 0.94) | 0.53 (0.33 – 0.86) |

7. *My other concern was also addressed in the limitations section of the discussion: 1) a main difference between symptomatic and asymptomatic infections clinically is that symptomatic infections are treated, whereas asymptomatic infections are not; differences in the risk of reinfection by homologous infections may in fact reflect the impact of treatment, rather than an impact of immunity. (suggest adding something along those lines in the limitations)*

RESPONSE: We have added the following to the Discussion:

Line 335: “Additionally, symptomatic infections were necessarily treated upon diagnosis, which could bias comparisons with asymptomatic infections which ended without treatment. Thus, differences in the risk of homologous reinfections may in fact reflect the impact of treatment rather than the impact of epitope type-specific immunity.”

8. *Text lines 114-118 and Figure 2. The authors show that presence of symptoms was not associated with risk of reinfection irrespective of sequence. Interestingly, having >50 mosquitos though appears to be counterintuitively associated with a significantly lower risk of reinfection (Fig 2B). The authors should provide some discussion of these results*

RESPONSE: We agree that the association of >50 mosquitoes with a lower risk of reinfection is interesting and puzzling. We first re-checked our code and confirmed that this variable is correctly coded; the “> 50 mosquitoes” category aligns well with the high transmission season, and likewise the “0-50 mosquitoes” category aligns nicely with the low season. Additionally interesting is that the association occurs in the intuitive direction (high transmission season is associated with increased risk of homologous reinfection) for epitope type-specific episodes. The change in the direction of the association could be due to the fact that during the high transmission season, super infection and serial re-infections can be observed with geno/epitope-typing; however, agnostic of genotypes, we are likely observing fewer, longer episodes during the high season. Although interesting, we have not included discussion of this phenomenon in our manuscript, as the analyses are not designed to measure the effect of transmission season on risk of reinfection. Generally, we interpret these associationa with caution, given that mosquito count was included in our models as a confounder for the primary exposure of symptomaticity and not as a comprehensive model of the association between mosquito count and reinfection; because of this, it is generally not recommended to interpret confounder effect estimates and infer risk (Westreich and Greenland *Am J Epi* 2013).

9. *Figure 2B: Please define reference groups for multivariate mixed effects model (specifically, define the reference group for >50 mosquitos – and is this a human biting rate? would help reader as opposed to having them look back at the methods) Also, in the methods this states “mosquitoes collected in the prior 14 days across study site”. Does that mean that this is not a marker of household level transmission but more a marker of village level transmission? Would specify (and cite a reference in methods; apologies if I missed that)*

RESPONSE: The mosquito level category was defined by the number of mosquitoes collected in the prior 14 days across all study sites and is meant to be a general proxy for transmission season. We have clarified this in the text. Additionally, we have added the reference groups to the forest plot labels in Figure 2b to provide more clarity and added text to the figure caption referring to the methods section. For Figure 4, we have added the reference groups for each variable referred to the methods section for variable definitions.

10. Line 181: “with parasites bearing…”

Line 196: “the risk of reinfection”

RESPONSE: Thanks for catching these typos. We have corrected them in the text.

We are much obliged for your consideration of our manuscript.

Warm regards,

Steve M Taylor MD MPH

Associate Professor of Medicine and Global Health

Duke University
